# Navigating the European Union Artificial Intelligence Act for Healthcare

**DOI:** 10.1038/s41746-024-01213-6

**Published:** 2024-08-12

**Authors:** Felix Busch, Jakob Nikolas Kather, Christian Johner, Marina Moser, Daniel Truhn, Lisa C. Adams, Keno K. Bressem

**Affiliations:** 1 School of Medicine and Health, Department of Diagnostic and Interventional Radiology, Klinikum rechts der Isar, TUM University Hospital, Technical University of Munich, Munich, Germany; 2 Department of Medical Oncology, National Center for Tumor Diseases (NCT), Heidelberg University Hospital, Heidelberg, Germany; 3 Else Kroener Fresenius Center for Digital Health, Technical University Dresden, Dresden, Germany; 4 Department of Medicine I, University Hospital Dresden, Dresden, Germany; 5 Johner Institut GmbH, Konstanz, Germany; 6 District Court, Cologne, Germany; 7 Department of Diagnostic and Interventional Radiology, University Hospital Aachen, Aachen, Germany; 8 School of Medicine and Health, Department of Cardiovascular Radiology and Nuclear Medicine, German Heart Center, TUM University Hospital, Technical University of Munich, Munich, Germany

**Keywords:** Health policy, Health care economics, Business, Technology, Industry

## Abstract

The European Union’s recently adopted Artificial Intelligence (AI) Act is the first comprehensive legal framework specifically on AI. This is particularly important for the healthcare domain, as other existing harmonisation legislation, such as the Medical Device Regulation, do not explicitly cover medical AI applications. Given the far-reaching impact of this regulation on the medical AI sector, this commentary provides an overview of the key elements of the AI Act, with easy-to-follow references to the relevant chapters.

## Introduction

The European Union (EU) has taken a critical step towards regulating artificial intelligence (AI) with the adoption of the AI Act by its 27 member states on March 13, 2024^1^. First proposed in April 2021 by the European Commission^2^, the AI Act emerged from the growing recognition of AI’s transformative potential and the need to address associated risks and ethical concerns, building on previous EU initiatives, including the 2018 Coordinated Plan on AI^3^ and the 2020 White Paper on AI^4^. The Council issued its position in December 2022^5^, followed by the European Parliament’s adoption of its negotiating position in June 2023^6^. After revising the draft, the final negotiations between the Commission and Council resulted in a provisional agreement in December 2023, which was endorsed by Member States in February 2024^1^.

As the world’s first comprehensive legal framework specifically on AI, the AI Act aims to promote human-centred and trustworthy AI while protecting the health, safety, and fundamental rights of individuals from the potentially harmful effects of AI-enabled systems (Article (Art) 1 (1)). The Act sets out harmonised rules for the placing on the market, putting into service and use of AI systems and has become binding law in all EU Member States 20 days after its publication in the Official Journal of the EU (published on July 12^th^ 2024), irrespective of existing national laws and guidelines on AI (Art 1 (2a), Art 113). Most parts of the regulation will take effect within 24 months, with prohibitions, i.e., bans on AI applications deemed to pose an unacceptable risk, taking effect already within 6 months (Art 113 (a–c)).

In its current form, the AI Act has a far-reaching scope, not only in the internal market but also extraterritorially, as it applies to all providers of AI systems in the EU market, regardless of where they are established or located (Art 2 (1a); see definitions of provider, deployer, and AI system in Table 1). In addition, the AI Act applies to providers and deployers of AI systems in third countries if the generated output is used in the Union (Art 2 (1c)). However, ‘output’ is not defined in the AI Act, and examples are vague, such as “*[…] predictions, content, recommendations, or decisions that can influence physical or virtual environments […]*” (Art 3 (1)). This suggests that any AI product could be subject to the AI Act if its output can be received in the Union, regardless of the provider’s or deployer’s intention or location. Finally, the AI Act also applies to “*[…] deployers of AI systems that have their place of establishment or are located within the Union […]*” (Art 2 (1b)). Therefore, deployers of AI systems within the EU, even if their models are not intended for the EU market, must comply with the AI Act regulations.

**Table 1:** Definitions of AI system, provider, deployer, and GPAI model and system in the EU AI Act

Term	Definition
AI system (Art 3 (1))	“[…] a machine-based system designed to operate with varying levels of autonomy, that may exhibit adaptiveness after deployment and that, for explicit or implicit objectives, infers, from the input it receives, how to generate outputs such as predictions, content, recommendations, or decisions that can influence physical or virtual environments […]”
Provider (Art 3 (3))	“[…] means a natural or legal person, public authority, agency or other body that develops an AI system or a general-purpose AI model or that has an AI system or a general-purpose AI model developed and places it on the market or puts the AI system into service under its own name or trademark, whether for payment or free of charge […]”
Deployer (Art 3 (4))	“[…] means a natural or legal person, public authority, agency or other body using an AI system under its authority except where the AI system is used in the course of a personal non-professional activity […]”
GPAI model (Art 3 (63))	“[…] an AI model, including where such an AI model is trained with a large amount of data using self-supervision at scale, that displays significant generality and is capable of competently performing a wide range of distinct tasks regardless of the way the model is placed on the market and that can be integrated into a variety of downstream systems or applications […]”
GPAI system (Art 3 (66))	“[…] an AI system which is based on a general-purpose AI model, that has the capability to serve a variety of purposes, both for direct use as well as for integration in other AI systems […]”

*AI* artificial intelligence, *Art* article, *EU* European Union, *GPAI* general-purpose artificial intelligence.

For the healthcare domain, the AI Act is particularly important, as other existing harmonisation legislation, such as the Medical Device Regulation (MDR; regulates products with an intended medical purpose on the EU market, classifying them from low-risk Class I (e.g., bandages) to high-risk Class III (e.g., implantable pacemaker)) or the In Vitro Diagnostic Medical Device Regulation (IVDR; regulates in vitro diagnostic devices with an intended medical purpose on the EU market, categorising them from Class A (low-risk, e.g., laboratory instruments) to Class D (high risk, e.g., products for detecting highly contagious pathogens such as Ebola)), do not explicitly cover medical AI applications. In addition, not all AI applications that can be adopted in the healthcare sector necessarily fall within the scope of the MDR or IVDR, e.g., general-purpose large language models (LLM) such as ChatGPT^2,3^.

Given the far-reaching impact of this regulation on the market, all stakeholders in the medical AI sector, including developers, providers, patients, and practitioners, can benefit from understanding its complex definitions, obligations, and requirements. Understanding the framework will help to clarify which models are covered by this regulation and the obligations that must be followed, ensuring that AI is implemented safely and responsibly, preventing potential harm, and ultimately driving innovation in medical AI. In this commentary, we navigate the most important aspects of the AI Act with a view to the healthcare sector and provide easy-to-follow references to the relevant chapters.

## Risk-based approach and innovation

**Fig. 1:**
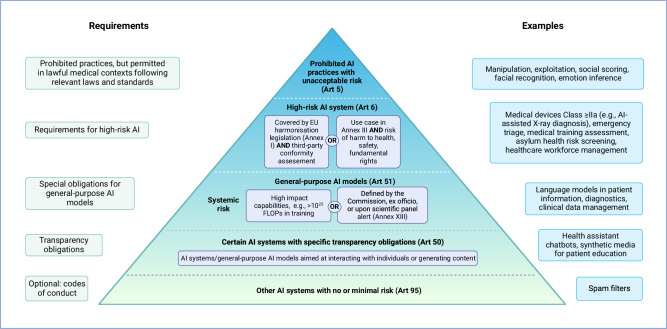
Schematic illustration of the risk-based approach of the EU AI Act, including relevant definitions, requirements, and examples. The pyramid structure illustrates the hierarchy of risk, with the most critically regulated or prohibited AI applications at the top and the least or unregulated AI applications at the bottom. This illustration does not necessarily correspond to the actual market share proportions of the individual risk categories. AI artificial intelligence, *Art* article, *EU* European Union. Created with biorender.com.

The AI Act follows a risk-based approach, focusing primarily on the prohibition of certain AI practices with unacceptable risks and the classification and obligations for high-risk AI systems and general-purpose AI (GPAI) models (Art 1 (2b–e)). A schematic illustration of the risk-based approach is presented in Fig. 1. Throughout the development process of the AI Act, a distinction has been made between AI systems, GPAI models, and their combination (GPAI systems) (see definitions in Table 1). The purpose of this distinction is to specify the particular obligations that GPAI models must always fulfil and to clarify responsibilities. While AI systems are supervised by national market surveillance authorities, the supervision of GPAI models and systems is carried out by a newly established AI Office at the EU level (Recital 116 (‘recitals’ are introductory statements that provide the context, background, or objectives of the legislation but do not contain binding legal provisions)).

Several measures have been taken to promote innovation and competitiveness and to facilitate the development of an AI ecosystem in the EU. First, the AI Act does not apply to AI systems developed and used solely for the purpose of scientific research or for personal, non-professional activities (Art 2 (6, 10)). Second, AI systems released under free and open-source licences are exempt from the AI Act requirements unless they use prohibited practices, are classified as high risk, or are subject to additional transparency obligations if they interact directly with individuals (Art 2 (12)). Furthermore, when these open-source systems are monetised, e.g., by providing paid technical support, they are again subject to the same regulations as their closed-source counterparts. This means that contrary to the hopes of open-source communities, the legal hurdles for open-source providers are essentially the same as for commercial providers.

To facilitate the streamlined market entry of commercial models, the AI Act offers the solution of regulatory sandboxes to be implemented at the national level within 24 months of the AI Act coming into force (Art 57 (1–17)). These are intended to enable the development, training, testing, and validation of AI systems in a controlled environment before they are brought to market and to prepare the provider to meet the necessary requirements. Nevertheless, the exact terms and conditions for these sandboxes have yet to be defined.

## Prohibited AI practices

The AI Act prohibits certain AI practices due to their unacceptable risks, including purposefully manipulative and deceptive practices, exploitation of vulnerabilities, biometric categorisation, social scoring, ‘real-time’ remote biometric identification, risk assessments for criminal offences, and facial recognition and emotion inference (Art 5 (1–8)). Non-compliance with these prohibitions can lead to severe penalties of up to 35 million EUR or 7% of annual turnover for companies (Art 99 (3)).

However, the AI Act provides exceptions for medical uses. For example, the prohibitions on manipulative and exploitative practices do not affect lawful practices in the context of medical treatment, such as psychological treatment for mental illness or physical rehabilitation, when carried out following applicable laws and medical standards and with the explicit consent of individuals or their legal representatives (Recital 29). Similarly, facial recognition and emotion recognition systems may be permitted for medical reasons (Art 5 (1f)).

## High-risk AI systems

There are two circumstances in which AI systems are classified as high risk: first, the AI system is intended to be used as a safety component of a product or is itself a product covered by the EU harmonisation legislation in Annex I, e.g., by the MDR or the IVDR, and is required to undergo a third-party conformity assessment under the relevant Union harmonisation legislation (Art 6 (1)). Here, it is important to note that compliance with the AI Act does not exclude compliance with all other relevant Union harmonisation legislation to be placed on the market. For example, in the MDR, only Class I medical devices do not require third-party conformity assessment^2^. Conversely, all medical AI products classified as Class IIa or higher must also fulfil the requirements of the AI Act for high-risk systems (e.g., AI-assisted medical image diagnosis)^7,8^.

The second circumstance concerns AI systems that are listed among the high-risk use cases in Annex III and pose a significant risk to the health, safety, or fundamental rights of natural persons (Art 6 (2, 3)). Here, examples of high-risk use cases in healthcare are systems intended for emotion recognition or emergency patient triage systems. For the second circumstance, an AI system can be exempted from the requirements if it is intended to perform a narrow procedural task, to improve the outcome of a previously performed human activity, to detect decision patterns or deviations from previous decision patterns, and is not intended to replace or influence the previously performed human assessment without proper human review, or is intended to perform a preparatory task for an assessment relevant to the purpose of the use cases listed in Annex III (Art 6 (3)). Therefore, common administrative tasks of AI systems in the medical field, such as medical text classification (e.g., ICD-10 coding) or structuring (e.g., structured radiology reporting), are unlikely to be classified as high risk unless they fall under the first circumstance. To further clarify this classification, the Commission will provide a more comprehensive list of practical examples of high-risk and non-high-risk use cases on AI systems within 18 months after the regulation enters into force (Art 6 (5)). Once an AI system is identified as high risk, certain requirements apply (see Table 2).

**Table 2:** Summary of EU AI Act requirements for high-risk AI systems

Requirement	Summary
1. Risk management system (Art 9)	• Continuous, iterative process throughout the product lifecycle • Identify and analyse known and foreseeable risks (for intended use and misuse) • Adopt appropriate, targeted risk management measures • Tests to determine the most suitable measures, including the potential impact on minors and vulnerable groups
2. Data and data governance (Art 10)	• Quality criteria for training, validation, and testing data sets for AI systems • Data governance practices focusing on design choices, collection processes, preparation operations, assumptions, bias detection/mitigation, and addressing data gaps • Data must be relevant, sufficiently representative, error-free, and tailored to the AI system’s intended purpose and target groups
3. Technical documentation (Art 11)	• General description: intended purpose, provider, version, hardware/software interaction, software/firmware versions, market/service forms, intended hardware, product component visuals, user-interface description, deployer instructions • Development and design process: development methods, design specifications, system architecture, data requirements and handling, human oversight assessment, pre-determined changes and performance, validation and testing procedures, cybersecurity measures • Monitoring and control details: performance capabilities and limitations, unintended outcomes and risk sources, human oversight measures, input data specifications
4. Record-keeping (Art 12)	• Automatic logging of events throughout the AI system’s lifecycle • Identification of situations that may result in the AI system presenting a risk or in a substantial modification, facilitation of post-market surveillance, operation monitoring
5. Transparency and provision of information to deployers (Art 13)	• Provision of characteristics, capabilities, and limitations of performance to enable deployers to understand how the AI system works, evaluate its functionality, and comprehend its strengths and limitations • Provision of user instructions
6. Human oversight (Art 14)	• Design and development of human–machine interface tools to enable effective human supervision to prevent or minimise risks • Empower users to understand the capabilities and limitations of the system, monitor its operation, be aware of and manage automation biases, interpret output accurately, make informed decisions about the use of the system, including disregarding or reversing its output, and safely intervene or stop the system when necessary
7. Accuracy, robustness, and cybersecurity (Art 15)	• Benchmarking accuracy and ensuring robust lifecycle performance • Resilience to errors, faults, inconsistencies, unauthorised changes via technical/organisational measures, redundancy, backups, safeguards against feedback loops and cybersecurity threats • Defence against data/model poisoning, adversarial examples, confidentiality attacks, and model flaws
8. Quality management system (Art 17)	• Appropriate to the size of the provider and sector, documentation through written policies, procedures, and instructions • Regulatory compliance strategies, design and development techniques, quality control and assurance processes, validation, verification, and testing procedures, application of technical specifications and standards, measures to ensure compliance with requirements not fully covered by harmonised standards
9. Corrective actions and duty of information (Art 20)	• If the AI system placed on the market is not in conformity with the AI Act, corrective actions must be taken
10. Authorised representatives (Art 22)	• For providers established in third countries, an authorised representative in the Union must be appointed, ensuring the provision of all necessary documentation and information and conformity with the AI Act
11. Fundamental rights impact assessment (Art 27)	• Certain deployers must assess the system’s impact on fundamental rights, covering how and why the system will be used, usage frequency and duration, affected individuals or groups, specific risks of harm and measures for risk mitigation, human oversight implementation, response plans for risk materialisation
12. Conformity assessment (Art 43)	• Compliance assessment of the quality management system, technical documentation, and post-market monitoring by the provider or a notified body
13. EU declaration of conformity (Art 47) and CE marking of conformity (Art 48)	• To indicate the conformity of the product with all applicable requirements set out in the relevant EU harmonisation legislation
14. EU database registration (Art 49)	• Providers of systems not covered by existing EU laws and those considering an Annex III system not high risk must register in a forthcoming Commission-managed EU database to increase transparency and facilitate Commission and Member State work
15. Post-market monitoring (Art 72)	• Proportional to the risks; collection and analysis of data on AI system performance • Implementation of a monitoring plan within technical documentation, guided by a Commission template
16. Reporting of serious incidents (Art 73)	• Reporting all serious incidents (i.e., serious harm to health, property, environment) to market surveillance authorities, investigating incidents, assessing risks, and implementing corrective actions

Non-exhaustive list of the key requirements in the AI Act. Articles not included refer to the cooperation and interaction with authorities or specific obligations for other stakeholders involved.

*AI* artificial intelligence, *Art* article, *CE* Conformité Européenne, *EU* European Union.

## GPAI models

GPAI models can generally be used as stand-alone high-risk AI systems or as components of other AI systems within any risk class. Irrespective of this, GPAI models must always fulfil certain requirements, as their capabilities allow for several downstream tasks (Recital 101). GPAI models are classified into presenting systemic risks, i.e., “*[…] a risk that is specific to the high-impact capabilities of general-purpose AI models, having a significant impact on the Union market due to their reach, or due to actual or reasonably foreseeable negative effects on public health, safety, public security, fundamental rights, or the society as a whole, that can be propagated at scale across the value chain […]*” (Art 3 (65)) or presenting no systemic risks, dependent on the capability of the model. A systemic risk is assumed if the model has high-impact capabilities, i.e., capabilities that match or exceed the capabilities recorded in the most advanced GPAI models (Art 51 (1a), Recital 111). A threshold defined in the AI Act is the cumulative amount of computation used for model training, measured in floating point operations (FLOPs), with a threshold of >10^25^ FLOPs being classified as presenting a systemic risk (Art 51 (2)). Notably, this threshold currently only affects very few models that are projected to scratch the surface of >10^25^ FLOPs, such as ChatGPT-4 or PaLM-2. However, the attribution of systemic risk can also be based on a decision of the Commission, ex officio, or following a qualified alert by the scientific panel based on criteria in Annex XIII (Art 51 (1b)). The key requirements to be met for GPAI models are displayed in Table 3. For GPAI models where a systemic risk can be assumed, additional requirements apply, including model evaluation, risk mitigation and management, and cybersecurity (Art 55 (1a–d)). Open-source GPAI models may be exempted from the listed transparency requirements if they do not pose a systemic risk (Art 53 (2)).

**Table 3:** Summary of EU AI Act requirements for general-purpose AI models

Requirement	Summary
1. Technical documentation and transparency information for downstream providers (Art 53 (1a, b))	• General model description: tasks, integration capabilities, use policies, release date, distribution, architecture, parameters, input/output modalities, formats, licence • Development and integration: technical requirements, design and training specifications, methodologies, key choices, optimisation goals, data details for training, testing, validation (type, provenance, curation, biases detection), computational resources, training time, known or estimated energy consumption • Open-source models may be exempted from these requirements if they do not pose a systemic risk
2. Copyright policy (Art 53 (1c))	• Implementation of an EU copyright-compliant policy
3. Training data transparency (Art 53 (1d))	• Publicly available summary about training content according to an AI Office template to be defined
4. Authorised representatives (Art 54 (1–5))	• For providers established in third countries, an authorised representative in the Union must be appointed, ensuring the provision of all necessary documentation and information and conformity with the AI Act
If a systemic risk is assumed:	
5. Model evaluation, risk mitigation and management, cybersecurity (Art 55 (1a–d))	• Evaluation using public protocols, tools, or other methodologies • Systemic risk assessment and mitigation, including adversarial testing • Ensuring the protection of cybersecurity

*AI* artificial intelligence, *Art* article, *EU* European Union.

To ensure that the requirements for GPAI are adequately met and maintained, codes of practice are envisaged to be developed by the industry with the participation of Member States and facilitated by the AI Office established by the European Commission (Art 56 (1–9)).

## Specific transparency obligations

Irrespective of the risk classification, all models, including GPAI, must fulfil additional transparency obligations if they are intended to interact with natural persons or to generate content, such as text, image, and video content (Art 50 (1, 2)). In the healthcare sector, this could be the case for virtual health assistants and chatbots. Here, transparency information for downstream providers must be provided, and the user must be informed about the use of AI (Art 50 (1)). Additionally, the output should be machine-readable and recognisable as artificially generated or manipulated (Art 50 (2)).

## AI systems not classified otherwise

AI systems, such as spam filters, that do not classify as high risk or GPAI and do not need to adhere to the specific transparency obligations are not subject to strict requirements under the AI Act. However, providers of these products are encouraged to voluntarily apply some or all of the mandatory requirements applicable to high-risk AI systems and to develop and adhere to codes of conduct along the elements foreseen in the European Ethical Guidelines for Trustworthy AI (Art 95 (2a)). In addition, minimising the impact of AI systems on environmental sustainability and promoting AI literacy, inclusive and diverse design of AI systems, and stakeholder participation should be key objectives of the codes of conduct (Art 95 (2b–d)).

## Implications for existing medical AI applications

Although the final version of the AI Act has been published in the Official Journal of the EU, the concrete impact on the future development of AI-enabled medical applications in the Union remains unclear as of August 2024. On the one hand, it is unlikely that the healthcare AI sector will be affected by prohibited practices under the AI Act due to the explicit exemptions for lawful medical purposes. However, given that ~75% of all commercial AI-enabled medical devices on the market are related to radiology^9^—all but one of which are classified as Class ≥IIa under the MDR^10^—most current solutions will be classified as high risk. As such, these devices must comply with the requirements for high-risk AI systems within 12 months of the AI Act coming into force (Article 113 (b)).

While the AI Act serves as so-called “horizontal” legislation spanning all AI-related industries, its effective implementation within pre-existing “vertical” legislations for each sector, such as the MDR or IVDR for the medical industry, has yet to be clarified^11^. As both MDR/IVDR and the AI Act are risk-based regulations, concerns arise because AI Act requirements, such as risk management, technical documentation, and conformity assessment, are already vertical standards that potentially intersect, conflict, or duplicate AI Act requirements, complicating the authorisation procedure^12^.

One solution could be third-party conformity assessment for higher risk medical AI products by the same independent notified bodies as for the MDR/IVDR to reduce the documentation burden and regulatory barrier. However, this becomes complicated for GPAI models, which will be regulated at the EU level by the AI Office and not by designated national authorities. Even if they are not explicitly intended for medical use, GPAI models must always meet certain requirements, and if they are used for medical purposes, such as LLM-enabled clinical reasoning and decision-making, they must also fulfil the MDR/IVDR and/or high-risk requirements. Therefore, although the final specifications and details of the implementation of the AI Act have yet to be defined, it is expected that the regulatory complexity and costs for most medical AI products in the EU market and beyond will rise. In particular, small and medium-sized enterprises with fewer resources are expected to suffer from the regulatory burden, weakening their market position^13^.

**Fig. 2:**
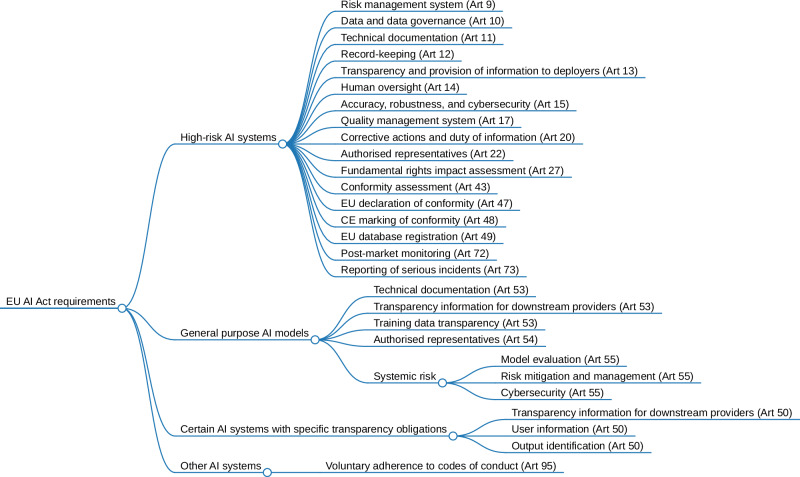
Hierarchical tree structure of the EU AI Act requirements for high-risk AI systems, general-purpose AI models, certain AI systems with specific transparency obligations, and AI systems that do not categorise otherwise. Non-exhaustive list of the key requirements in the AI Act. Articles not included refer to the cooperation and interaction with authorities or specific obligations for other stakeholders involved. AI artificial intelligence, Art article, CE Conformité Européenne, EU European Union. Created with markmap.js.org.

In conclusion, the AI Act, as the first-ever comprehensive legal framework dedicated to AI, aims to ensure the safe and fair development and implementation of AI across a range of industries, including healthcare. This comment provides a comprehensive and accessible guide to the most important aspects of the AI Act, including practical examples from the medical field. An overview of all requirements for each classification set out in the AI Act is given in Fig. 2.

Beyond the EU’s borders, the AI Act may also impact the global market by setting high standards for the development and use of AI that can be followed and adopted by other national or international regulators and authorities. However, the rapid development of AI technologies will require the ongoing reassessment and refinement of such regulations. Failure to adopt these frameworks quickly could lead to unequal market opportunities or significant consumer risks. It remains to be determined how this can be adequately managed within the regulatory landscape of AI.

## References

[1] European Parliament. (2024, accessed 10 April 2024). https://www.europarl.europa.eu/doceo/document/TA-9-2024-0138_EN.pdf.

[2] European Commission. (2021, accessed 14 June 2024). https://eur-lex.europa.eu/legal-content/EN/TXT/?uri=celex%3A52021PC0206.

[3] European Commission. (2018, accessed 14 June 2024). https://eur-lex.europa.eu/legal-content/EN/TXT/?uri=CELEX%3A52018DC0795.

[4] European Commission. (2020, accessed 14 June 2024). https://eur-lex.europa.eu/legal-content/EN/TXT/PDF/?uri=CELEX:52020DC0065.

[5] Council of the European Union. (2022, accessed 15 June 2024). https://data.consilium.europa.eu/doc/document/ST-14954-2022-INIT/en/pdf.

[6] European Parliament. (2023, accessed 15 June 2024). https://www.europarl.europa.eu/doceo/document/TA-9-2023-0236_EN.pdf.

[7] European Parliament, Council of the European Union. (2017, accessed 10 April 2024). https://eur-lex.europa.eu/legal-content/EN/TXT/?uri=CELEX%3A32017R0745.

[8] European Parliament, Council of the European Union. (2017, accessed 10 April 2024). https://eur-lex.europa.eu/eli/reg/2017/746/oj.

[9] European Commission, EUDAMED—European Database on Medical Devices. (2024, accessed 17 June 2024). https://ec.europa.eu/tools/eudamed/.

[10] Radiology Health AI Register. (2024, accessed 17 June 2024). https://radiology.healthairegister.com/products.

[11] Gilbert, S. The EU passes the AI Act and its implications for digital medicine are unclear. *NPJ Digit. Med.* **7**, 135 (2024). 38778162 10.1038/s41746-024-01116-6 PMC11111757

[12] MedTech Europe. *Medical Technology Industry Perspective on the Final AI Act* (2024, accessed 15 June 2024). https://www.medtecheurope.org/wp-content/uploads/2024/03/medical-technology-industry-perspective-on-the-final-ai-act-1.pdf.

[13] Gassauer, J. & Burch, G. F. *The Potential Impact of the European Commission’s Proposed AI Act on SMEs*. (2023, accessed 15 June 2024). https://www.isaca.org/resources/isaca-journal/issues/2023/volume-2/the-potential-impact-of-the-european-commissions-proposed-ai-act-on-smes.

